# Plasma Brain-Derived Neurotrophic Factor Levels in First-Episode and Recurrent Major Depression and before and after Bright Light Therapy in Treatment-Resistant Depression

**DOI:** 10.3390/biom13091425

**Published:** 2023-09-20

**Authors:** Biljana Kosanovic Rajacic, Marina Sagud, Drazen Begic, Matea Nikolac Perkovic, Anja Dvojkovic, Lana Ganoci, Nela Pivac

**Affiliations:** 1 Department for Psychiatry and Psychological Medicine, University Hospital Centre Zagreb, 10000 Zagreb, Croatia; bkosanov@gmail.com (B.K.R.); marinasagud@mail.com (M.S.); drazen.begic@mef.hr (D.B.); 2 School of Medicine, University of Zagreb, 10000 Zagreb, Croatia; 3 Laboratory for Molecular Neuropsychiatry, Division of Molecular Medicine, Ruder Boskovic Institute, 10000 Zagreb, Croatia; mnikolac@irb.hr; 4 University Psychiatric Hospital Vrapce, 10090 Zagreb, Croatia; anja.maravic@gmail.com; 5 Department of Laboratory Diagnostics, Division for Pharmacogenomics and Therapy Individualization, University Hospital Centre Zagreb, 10000 Zagreb, Croatia; lana.ganoci@gmail.com; 6 University of Applied Sciences Hrvatsko Zagorje Krapina, 49000 Krapina, Croatia

**Keywords:** brain-derived neurotrophic factor, bright light therapy, major depressive disorder, plasma concentration, remission, treatment-resistant depression, treatment response

## Abstract

Brain-derived neurotrophic factor (BDNF) is implicated in the etiology and treatment response in major depressive disorder (MDD). However, peripheral BDNF concentrations have not been compared across different MDD stages. Bright light therapy (BLT) offers some potential in treatment-resistant depression (TRD), but its effects on BDNF levels are unknown. This study included a cross-sectional analysis of plasma BDNF concentration in females with TRD, unmedicated MDD patients, and healthy controls (HC), and measurements of longitudinal BLT effects on plasma BDNF levels in TRD patients. The present study included 55 drug-naïve, first-episode patients, 25 drug-free recurrent-episode MDD patients, 71 HC participants, and 54 TRD patients. Patients were rated by Hamilton Depression Rating Scale (HAMD)-17 and the Montgomery–Åsberg Depression Rating Scale (MADRS). Patients with TRD received BLT during 4 weeks. The total HAMD-17 and MADRS scores decreased following BLT. All patient groups had lower plasma BDNF than HC, but BDNF levels did not differ between first- and recurrent-episode BDNF patients and TRD patients before or after BLT. However, responders and remitters to BLT had higher post-treatment plasma BDNF concentrations than patients who did not achieve response or remission. The changes in plasma BDNF levels may be candidates for biomarkers of treatment response to BLT in TRD patients.

## 1. Introduction

Antidepressants are the first-line treatment for major depressive disorder (MDD). However, between one third [1,2,3,4] and one fifth [5] of MDD patients worldwide suffer from treatment-resistant depression (TRD). Compared to patients who responded well to treatment, patients with TRD have more severe symptoms [4], a higher number of life-time suicide attempts [1], a heightened risk of suicide mortality [5], lower quality of life, greater functional impairment [6], and, consequently, represent a significant financial burden [2].

Pharmacological options for TRD include augmentation with new-generation antipsychotics [7,8], mood stabilizers such as lithium or lamotrigine, thyroid hormones [7,9], and N-methyl-D-aspartate-targeting agents [9] including esketamine [10]. Other interventions comprise various modalities of transcranial magnetic stimulation (TMS) [11], electroconvulsive therapy (ECT), transcranial direct current stimulation, deep brain stimulation, and vagus nerve stimulation [12]. However, such treatments are limited by the adverse events [7,8,10] and neuromodulation therapies are not widely available.

Another option for MDD is bright light therapy (BLT). It has distinct mechanism of action from all other treatments, i.e., light affects retinal ganglion cells, inducing a cascade of events in several pathways related to the etiology of depression, such as suprachiasmatic nucleus and lateral habenula [13]. In several small, noncontrolled studies, BLT addition to current pharmacotherapy has shown some efficacy in TRD, either alone [14,15] or in combination with other chronotherapeutic interventions [16,17,18] or with repetitive TMS (rTMS) [19]. In a randomized trial in patients with nonseasonal unipolar or bipolar depression, BLT was more efficacious than placebo in a subgroup of participants who had treatment resistance [20]. In another randomized trial on unipolar and bipolar TRD patients, BLT in combination with rTMS was more efficacious that rTMS alone [21]. 

Bright white light represents a mixture of the spectrum of wavelengths most similar to daylight. BLT is most often applied in the early morning. However, a small number of depressed patients may benefit from the evening treatment, according to their specific phase shift of the circadian rhythm. Sophisticated fluorescent lamps without emitting ultraviolet (UV) rays and with a light intensity of 10,000 lx are most often used for the significant therapeutic effect, although there are lamps that emit light of a lower intensity.

Brain-derived neurotrophic factor (BDNF), which is the most abundant neurotrophin and is involved in neuroplasticity and neurotransmission, also has an important role in the etiology and treatment of depression [22]. Apart from the brain, it is also present in the blood, platelets, serum, and plasma. Multiple lines of evidence demonstrated lower peripheral BDNF levels in patients during depressive episode than euthymic patients or healthy controls [23,24]; however, it is not clear if such levels differ between first or recurrent episode, and TRD patients. The effects of various antidepressant interventions on peripheral BDNF levels were also extensively investigated [25], but very few studies address the influence of BLT on BDNF. In a preclinical trial using rats, bright light increased BDNF protein levels in the hippocampus and induced hippocampal neurogenesis [26]. In humans, an increase in serum and a decrease in saliva BDNF levels was reported in healthy women after 3 weeks of light therapy [27], while the other study showed no effects of light therapy on serum BDNF levels in older individuals [28]. Interestingly, serum BDNF concentrations correlated with the number of sunlight hours in healthy participants and psychiatric patients, including those with MDD [29]. A recent meta-analysis reported the overall increase in peripheral BDNF levels following different treatment strategies in TRD patients [30], but the impact of BLT on circulatory BDNF levels in TRD patients (and even in MDD patients) is completely unknown.

The aim of the present study was to (1) compare plasma BDNF levels between medicated patients with TRD, drug-free MDD patients (in the first or recurrent episode), and in healthy controls (HC), and to (2) examine the effects of BLT on plasma BDNF levels in TRD patients. The hypothesis was that plasma BDNF concentration will be decreased in patients with different forms of depression compared to healthy subjects, and that treatment (i.e., BLT) will increase plasma BDNF concentration in TRD patients.

## 2. Materials and Methods

### 2.1. Participants

All participants were recruited at the Department of Psychiatry and Psychological Medicine, University Hospital Centre Zagreb. The diagnosis of MDD for all patients was confirmed by a structured clinical interview based on the DSM-5 criteria [31].

Three groups of female patients with depression were included: medicated patients with TRD, drug-free patients with MDD (which consisted of two subgroups: patients in the first episode and patients in the recurrent episode of MDD), and we also included female healthy controls. 

The inclusion criteria for all participants were as follows: only females were recruited, aged 18 to 70 years, who signed informed consent document approved by the Ethics Committees of the University Hospital Centre Zagreb and the Medical School of the University of Zagreb.

The inclusion criteria for the drug-free MDD group were being in an acute depressive episode, either drug-naïve (in the first episode of MDD), or not receiving antidepressants for those with recurrent MDD, for at least 4 weeks (or 6 weeks in case of fluoxetine), with a minimal Hamilton Depression Rating Scale (HAMD)-17 [32] score of 15, without documented treatment resistance to previous antidepressants.

Patients were recruited in the TRD group if they met the definition of a lack of response to at least two unsuccessful antidepressant trials of adequate duration and dose during the current episode [33], and being on the same antidepressant therapy for at least 4 weeks before enrolment. 

The inclusion criteria for healthy control women were as follows: individuals without current or previous psychiatric disorders, and also without known current severe somatic or neurological conditions.

The exclusion criteria for all patients involved were previous craniocerebral injury or surgery; alcohol or other substance dependence in the previous 6 months; psychiatric comorbidities, including seasonal affective disorder, bipolar disorder, and/or schizophrenia spectrum disorders; current suicidality; severe and unstable somatic and neurological comorbidities, such as malignancy, epilepsy, dementia, cerebral aneurysm, immune disease, or acute infectious disease; and pregnancy and breastfeeding. To reduce the possible factors influencing BDNF concentration, we warned all participants to avoid physical exercise, tobacco, and alcohol consumption during the 24 h prior to the experiment. 

Additional exclusion criteria for TRD patients involved severe eye conditions such as glaucoma, retinopathies, and eye infection, electroconvulsive therapy (ECT) application up to 3 months before the study, undergoing individual psychotherapy, and an earlier application of phototherapy. The use of antipsychotics and mood stabilizers was not allowed. Anxiolytics and short supportive psychotherapy support as an integral part of the psychiatric examination was allowed.

### 2.2. Study Design

All patients completed the Montgomery–Åsberg Depression Rating Scale (MADRS) [34] and HAMD-17. Patients with drug-free MDD (either in the first-episode or in the recurrent-episode group) had single assessments and single BDNF measurements. The study protocol is shown in Table 1. 

Patients with TRD were assessed at inclusion and after 4 weeks of BLT. Clinical response was defined as a ≥50% reduction from the baseline in the HAMD-17/MADRS score [35,36], following 4 weeks of treatment. According to the same schedule, before and after 4 weeks of BLT, the patients had fasting blood taken in a test tube at 8:30 a.m. for the purpose of measuring plasma BDNF concentrations.

### 2.3. Bright Light Therapy

Patients diagnosed with TRD received BLT as an add-on therapy in the morning hours according to the schedule, for 4 days per week, for 4 weeks, lasting 20 min a day. At the same time, they continued to take their antidepressant therapy, to which they had not responded well at least 4 weeks before inclusion into the study. BLT was performed by a psychiatrist with a Bioptron pro 1 lamp manufactured by Zepter, Wollerau, Switzerland, with a filter diameter of 11 cm, and a light intensity of 10,000 lx at a distance of 30 to 40 cm. The Bioptron pro 1 lamp is a sophisticated device for professional medical use, which emits light similar to the part of the electromagnetic spectrum naturally produced by the Sun but without UV radiation [37,38].

Six TRD patients did not complete the 4 weeks of BLT, for the following reasons: 2 of them experienced worsening of the symptoms, 2 reported an impression of BLT ineffectiveness and refused further applications, 1 had acute somatic illness, and 1 reported headaches during BLT treatment. 

### 2.4. Blood Sampling and Plasma BDNF Measurement

Sampling was performed after the overnight fast, at 8.30 a.m., during routine laboratory visits. A total of 3–4 mL of blood was sampled from all subjects and was immediately processed. Tubes with citrate anticoagulant were used—a container with a blue cap (Becton, Dickinson and Company, Franklin Lakes, NJ, USA). To obtain platelet-poor plasma samples, the blood samples were centrifuged twice. The first centrifugation lasted 15 min at 1000× *g*, and the second centrifugation lasted 10 min at 10,000× *g*. Plasma samples were separated with an automatic pipette into polypropylene containers (Eppendorf, Hamburg, Germany) and stored in a freezer at −20 °C until the BDNF concentration was determined. 

Concentrations of total BDNF in platelet-poor plasma were determined by enzyme-linked immunosorbent assay (ELISA) using the commercial reagent kit Quantikine^®^ ELISA Human BDNF (R&D Systems Europe Ltd., Abingdon, UK), as previously described and validated. Measurements were performed in triplicate for all samples. 

Plasma samples were thawed out and all reagents were prepared at room temperature. A total of 20 mL of wash buffer concentrate and 480 mL of distilled water were measured using a beaker and mixed in a glass. The color reagents A and B were mixed in equal proportions 15 min before use and were protected from light. Calibrator Diluent RD6P was added to the bottle of Human Free BDNF Standard up to the mark and dissolved by gentle stirring for 15 min. The stock solution of the BDNF standard was obtained at a concentration of 4000 pg/mL. Further dilutions were prepared in polypropylene containers by adding 300 µL of the mother standard and 300 µL of Calibrator Diluent RD6P, so 6 standards were obtained with concentrations from 62.5 pg/mL to 2000 pg/mL, according to the scheme. A total of 100 μL of Assay Diluent RD1S was pipetted into each well of the microtiter plate. In addition, 50 μL of standard, control, or sample was pipetted into each well. The microtiter plate was covered with an adhesive cover and incubated for 2 h at room temperature. Then, 100 μL of Human Free BDNF Conjugate solution was added to each well. The microtiter plate was covered with a new adhesive cover and incubated for 1 h at room temperature. Using a multichannel pipette, the contents of each well were aspirated, and they were washed by adding 400 mL of washing buffer to each well. The procedure was repeated 3 times, and after the last washing, the microtiter plate was thoroughly dried by turning it over and patting it on clean tissue. A total of 200 μL of substrate solution was pipetted into each well. Protected from light, the microtiter plate was incubated for 30 min at room temperature. In addition, 50 μL of stop solution was pipetted into each well. With gentle tapping, thorough mixing was ensured until the color of the wells changed from blue to yellow. Over 30 min, the optical density was read on a Dynex MRX reader (Dynex Technologies Inc., Sullyfield Circle Chantilly, VA, USA) at 450 nm, with wavelength correction at 540 nm. The reader then automatically generated a calibration curve and read the BDNF protein concentrations. 

### 2.5. Statistics 

Data processing and the statistical evaluation were performed using the SAS software (SAS Institute, Cary, NC, USA). In all statistical analyses, the α significance level was set at 0.05, and all tests used were two-tailed. The Kolmogorov–Smirnov test was used to assess the normality of the demographic and clinical data. Due to deviation from the normal distribution, all clinical and sociodemographic parameters, and BDNF plasma concentration, are presented as the median and the range between the smallest (min) and largest (max) value. When comparing two independent groups of data, the Mann–Whitney U test was used, and in the case of comparing several independent groups, Kruskal–Wallis ANOVA by ranks was used. In the case of the comparison of dependent groups, Wilcoxon rank test of dependent samples (Wilcoxon test) was used. The distribution of categorical data was evaluated using the Chi-square (χ^2^) test. Spearman’s correlation coefficient (ρ) was used to analyze the potential association of HAMD-17 and MADRS scores with the BDNF plasma concentration.

Multiple linear regression analysis was used to evaluate the possible influence of age and smoking status on plasma BDNF concentration in patients diagnosed with depression and healthy controls. 

Analysis of covariance (ANCOVA) was used to compare BDNF concentration after phototherapy between groups of subjects, who were divided according to their response to therapy or achieved remission, using the baseline value of plasma BDNF as a covariate for the model correction. 

Prior to ANCOVA, log10 data transformation of plasma BDNF was performed in order to deal with the deviation from the normal distribution. 

The fold change in BDNF plasma concentration was calculated as the ratio (B/A) of the BDNF plasma concentration after therapy (B) and BDNF plasma concentration before therapy (A).

## 3. Results

### 3.1. Subject Characteristics

This study included three groups of female patients with depression: medicated patients with TRD (N = 54); drug-free patients with MDD (N = 80), which consisted of two subgroups, 55 patients in the first episode and 25 patients in the recurrent episode of MDD; and female healthy controls (N = 71). Among 66 TRD candidates, 60 patients met the criteria during screening process. Therefore, six patients were not included: three due to malignant disease, two due to taking mood stabilizers, and one due to retinopathy. The demographic and clinical features of subjects included in the study are presented in Table 2. Groups of subjects diagnosed with various forms of depression (first-episode MDD vs. recurrent-episode MDD vs. TRD) did not differ in the proportion of subjects with a positive family history of depression (*p* = 0.427), and in the number of subjects who had a positive suicide attempt history (*p* = 0.483). Also, these three patient groups had similar HAMD-17 (*p* = 0.246) and MADRS (*p* = 0.373) scores. However, there was a significant difference in the percentage of smokers between three groups of MDD patients and healthy control subjects (*p* = 0.001), all due to the higher percentage of smokers in the TRD group (Table 2). The results also indicate a significant difference in age between the four subject groups (*p* < 0.001). Patients with first-episode MDD were significantly younger than the other groups, as expected (Table 2).

### 3.2. Plasma BDNF Concentration in MDD Subjects

Multiple linear regression analysis was used to evaluate the possible influence of age and smoking status on plasma BDNF concentration in patients diagnosed with depression and healthy controls. Plasma BDNF concentration was set as the dependent variable, and age and smoking as independent variables. The obtained model was not significant (F(2, 161) = 2.94; *p* = 0.056; Radj2 = 0.023). This lack of a significant association was due to the lack of significant effect of smoking (*p* = 0.140) and age (*p* = 0.073) on plasma BDNF concentration. Therefore, in the further analyses, participants were not subdivided according to age or smoking.

**Figure 1:**
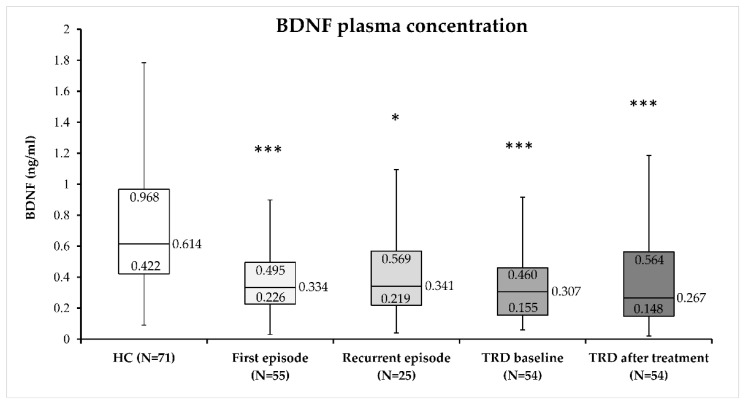
Plasma BDNF concentration in healthy subjects (HC), patients with first or recurrent-episode MDD, and subjects with treatment-resistant depression (TRD) before and after 4 weeks of treatment with BLT: *** *p* < 0.001 vs. HC; * *p* < 0.05 vs. HC (Kruskal–Wallis ANOVA followed by the Dunn’s post-hoc method). Median value and quartile (Q1 and Q3) values for each group are indicated in the figure.

Plasma BDNF concentrations were compared between subjects diagnosed with MDD (first-episode MDD, recurrent-episode MDD, TRD before, and TRD after 4 weeks of phototherapy) and healthy control subjects (H = 34.32; *p* < 0.001). Plasma BDNF concentration did not differ significantly between patients with first-episode MDD, recurrent-episode MDD, TRD before, and TRD after 4 weeks of phototherapy. However, plasma BDNF concentration was significantly higher in healthy subjects compared to different groups of subjects diagnosed with MDD (Figure 1).

### 3.3. Plasma BDNF Concentration in TRD Patients before BLT

To evaluate the possible effect of suicidal behavior on plasma BDNF concentration in TRD patients, we compared baseline plasma BDNF in patients who tried to attempt suicide (0.53 ng/mL) vs. TRD patients without suicidal attempt (0.30 ng/mL), and plasma BDNF concentration did not differ significantly (U = 77.00; *p* = 0.185; Mann–Whitney test). To confirm the results from the multiple linear regression analysis in patients with TRD, baseline plasma BDNF concentration was compared in TRD smokers (0.24 ng/mL) vs. TRD nonsmokers (0.31 ng/mL), and plasma BDNF concentration was not significantly different (U = 0.392; *p* = 0.149; Mann–Whitney test).

At baseline, HAMD-17 (ρ = 0.045; *p* = 0.746) and MADRS (ρ = −0.031; *p* = 0.822) scores were not significantly correlated with plasma BDNF concentration. Other clinical variables, such as the length of treatment of MDD (ρ = −0.062; *p* = 0.656), the number of depressive episodes (ρ = 0.059; *p* = 0.673), the length of the last depressive episode (ρ = −0.132; *p* = 0.342), the number of hospitalization (ρ = −0.115; *p* = 0.407), or the number of used antidepressant medications in the therapy (ρ = 0.039; *p* = 0.781), were also not significantly correlated (Spearman’s coefficient of correlation) with plasma BDNF concentration before BLT.

### 3.4. BLT and Plasma BDNF Concentration in TRD Patients

The effect of BLT in TRD was assessed by comparing HAMD-17 and MADRS scores before and after 4 weeks of treatment in TRD patients. The total HAMD-17 (*p* < 0.001) and MADRS (*p* < 0.001) scores decreased significantly after 4 weeks of treatment compared to baseline scores in TRD patients (Table 3). 

**Figure 2:**
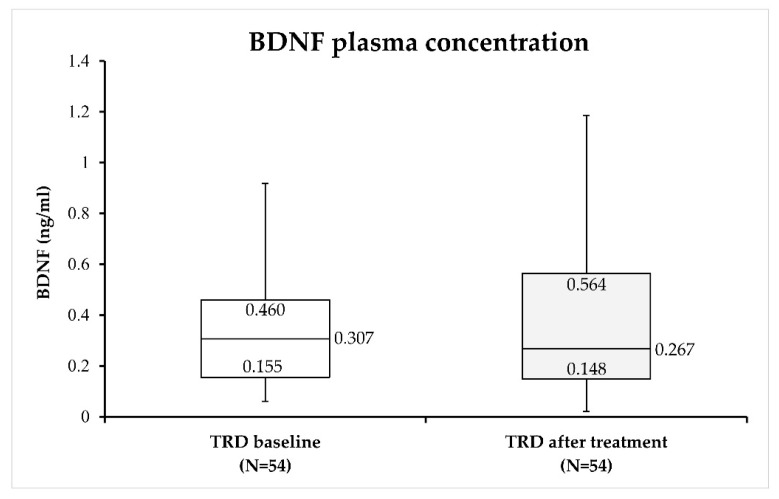
Plasma BDNF concentration in subjects with treatment-resistant depression (TRD) before and after 4 weeks of treatment with BLT. Median value and quartile (Q1 and Q3) values for each group are indicated in the figure.

The comparison of plasma BDNF concentration before and after 4 weeks of BLT revealed no significant changes (W = 759.00; *p* = 0.887) in the BDNF concentration after BLT compared to BDNF values before BLT in TRD patients (Figure 2).

In order to further examine the possible association of the BDNF plasma concentration with the response to BLT, subjects with TRD were divided into two groups: those who had a good response to BLT (a decrease in HAMD-17 or MADRS scores ≥50%, i.e., responders) and those who did not achieve this reduction (i.e., nonresponders). ANCOVA was used to assess the difference in BDNF plasma concentrations between responders and nonresponders to BLT, using the baseline value of plasma BDNF as a covariate for the model correction. Prior to ANCOVA, log10 data transformation of plasma BDNF was performed in order to deal with the deviation from the normal distribution. ANCOVA results revealed a significant difference in the post-treatment plasma BDNF concentration in subjects with TRD who responded well to BLT compared to those who did not (F = 4.05; df = 2.51; *p* = 0.023), since responders had a higher plasma BDNF concentration than nonresponders. A similar effect (i.e., increased plasma BDNF concentration in responders vs. nonresponders) was observed when subjects were divided according to MADRS scores after the treatment (F = 3.45; df = 2.51; *p* = 0.039). The average fold change in BDNF plasma concentration after BLT was 2.21 in responders and 1.25 in nonresponders, when using HAMD-17 scores to assess response to treatment. When MADRS scores were used to define responders and nonresponders to BLT, the average fold change in the BDNF plasma concentration was 2.13 in responders to phototherapy and 1.37 in nonresponders. These results indicate a greater increase in plasma BDNF concentration in TRD patients who responded well to BLT, compared to nonresponders.

The HAMD-17 and MADRS scores were also used to assess the achieved remission in patients with TRD after treatment with BLT. Remission was defined as HAMD-17 scores ≤ 7 [39], or MADRS scores ≤ 10 [40] after 4 weeks of phototherapy. In order to examine the association between BDNF plasma concentration and remission, the TRD subjects were divided into those who achieved remission (i.e., remitters) and those who did not achieve remission (i.e., nonremitters). The comparison was made using ANCOVA, with the baseline concentration of plasma BDNF as a covariate for the model correction. Due to the deviation from normal distribution, log10 data transformation of plasma BDNF was performed before ANCOVA. The results of ANCOVA revealed a significant difference (i.e., increased plasma BDNF concentration in remitters) in post-treatment plasma BDNF concentration in subjects who achieved remission (post-treatment HAMD-17 scores ≤ 7) compared to TRD patients treated with BLT who did not achieve remission, i.e., nonremitters (F = 5.71; df = 2.51; *p* = 0.006). A similar result (higher plasma BDNF concentration in remitters vs. nonremitters) was visible when patients with TRD were divided according to the post-treatment MADRS scores (F = 3.59; df = 2.51; *p* = 0.035). The average fold change in BDNF plasma concentration after BLT was 4.00 in patients who achieved remission and 1.32 in those who did not achieve remission, after using HAMD-17 to assess remission. When the MADRS score was used to define remission, the average fold change in BDNF plasma concentration was 2.77 in patients who achieved remission and 1.41 in those who failed to achieve remission. Therefore, a significantly higher plasma BDNF concentration was detected in TRD remitters vs. TRD nonremitters after BLT. These results indicate a greater increase in plasma BDNF concentration in TRD patients who went into remission after 4 weeks of BLT, compared to TRD subjects who failed to achieve remission.

## 4. Discussion

The results of the present study show that (1) TRD patients had lower plasma BDNF concentrations than healthy controls, but their BDNF concentration was similarly decreased in other drug-free MDD patients; (2) TRD patients continued to have low plasma BDNF concentrations after BLT; however, (3) while BLT did not affect BDNF concentration in the entire TRD group, responders and remitters with TRD had significantly higher post-treatment plasma BDNF concentrations than nonresponders and nonremitters, respectively. Therefore, we confirmed our hypothesis that patients with various forms of depression have lower plasma BDNF concentrations than healthy control subjects, and we partially confirmed the expectation that good treatment response and remission was associated with higher plasma BDNF concentrations in TRD patients.

### 4.1. Plasma BDNF Concentration in MDD Patients

Our findings implicate decreased plasma BDNF levels in MDD patients, which were similarly low across the drug-free recurrent group, the drug-naïve first-episode group, and medicated TRD patients. This finding is in general agreement with the majority of other studies that revealed lower plasma BDNF levels in unmedicated MDD participants [41] and first-episode depression patients [42,43] than in healthy controls, but some studies found similar plasma BDNF values between first-episode, drug-naïve MDD patients and healthy controls [44,45]. In contrast to our findings, one study found lower plasma BDNF levels in recurrent-episode MDD patients than first-episode patients [46]. The discrepancies may arise from different clinical features that may impact plasma BDNF concentration. In the cited study, unlike in our trial, patients in both groups had higher severity of symptoms and psychotic patients were included [46]. For example, psychotic symptoms in drug-naive first-episode psychotic patients were associated with reduced plasma and cerebrospinal fluid BDNF concentration, compared to BDNF concentration in healthy controls [47]. On the other hand, there is also evidence of a higher plasma BDNF levels in psychotic than nonpsychotic MDD patients [46]. We were not able to find a study which compared BDNF between patients in first or recurrent MDD, TRD patients, and controls. However, a meta-analysis reported similar (lower) plasma BDNF in patients with MDD and bipolar depression, compared to euthymic patients [23]. Notwithstanding some differences across studies, we hypothesize that different forms of depression, primarily in drug-free patents, may present with a similar degree of low plasma BDNF compared to healthy individuals. 

In our study, the baseline plasma BDNF concentration was not significantly correlated with HAMD-17 or MADRS scores, or with clinical measures such as the length of treatment of MDD, the number of depressive episodes, the length of the last depressive episode, the number of hospitalizations, or the antidepressant medication used. These data are in line with the lack of association found between blood BDNF levels and HAMD-17 or the Hamilton Rating Scale for Anxiety scores in MDD patients [48,49] or with depressive symptoms in TRD reported in a meta-analysis [30]. In contrast to our data in TRD, in a study including only 39 patients with MDD, plasma BDNF levels were negatively correlated with the severity of depressive and anxiety symptoms [41]. 

Another potential confounder may be previous suicidal behavior. More specifically, among medicated recurrent-episode MDD patients, plasma BDNF was higher in participants with positive, compared to those with negative, suicide attempt history, but one third of this sample had previous suicide attempts [50] compared to less than 10% in our TRD patients. However, in our study, suicidal behavior, i.e., previous suicidal attempt, did not significantly alter plasma BDNF concentration in TRD patients and BDNF concentration did not differ between suicidal and nonsuicidal TRD patients. In contrast, a meta-analysis reported lower plasma BDNF levels among psychiatric patients who attempted suicide, including those with MDD, than nonattempters [51]. Differences between studies might be induced by the inclusion of TRD patients (our study) compared to MDD patients [51].

A recent meta-analysis showed the lower circulatory BDNF levels in TRD compared to healthy controls, but the vast majority of studies in that review elucidated serum BDNF [30]. In line with our data, one study assessed plasma BDNF in TRD patients, and found lower levels compared to healthy subjects [52]. Our results expand on those findings showing that reduced BDNF levels were similar to values in drug-free patients with depression. The results showing lower BDNF levels in TRD are slightly intriguing, given that all our patients were medicated, and antidepressants were expected to normalize BDNF levels [53]. However, no significant differences in serum BDNF levels in TRD patients treated with esketamine or ketamine were reported [54].

However, not all antidepressants decrease BDNF levels, since it has been reported that escitalopram [55], fluoxetine, and fluvoxamine [56] treatments do not affect plasma BDNF concentration, and plasma BDNF was similar to healthy controls only in euthymic MDD patients [23]. 

Our present findings might suggest that plasma BDNF is low in untreated patients with depression, normalized in remitted patients, but still low in those who did not respond to treatment. Such an assumption makes BDNF a potential state marker in depression, but many potential confounders need to be targeted in future studies, such as BDNF gene polymorphisms, life-style habits, severity and specific features of depression, BDNF assessment methods, and detection of mature BDNF or pro-BDNF [57].

### 4.2. Plasma BDNF Concentration after BLT

Our TRD patients had a good overall response to BLT add-on treatment, which is in line with other studies [14,15,20]. However, our participants with TRD had moderately severe symptoms, as reflected from the HAMD-17 and MADRS scores, suggesting that our findings cannot be generalized to individuals with a more severe psychopathology. 

Our results only implicate the changes in BDNF levels after BLT in patients who experienced clinical improvement. While an overall increase in BDNF levels was detected after TRD treatment in other trials, such findings were not unambiguous, and no association was detected between peripheral BDNF and antidepressant effects [30]. However, it should be stressed that this meta-analysis predominantly assessed serum BDNF [30]. Serum and plasma represent separate compartments, with different BDNF dynamics. Plasma BDNF was significantly positively correlated to cerebrospinal fluid BDNF concentration, and therefore the authors suggested that plasma BDNF levels reflect the brain changes in BDNF levels [47]. While plasma and serum BDNF were correlated in healthy individuals [58] or in drug-free schizophrenia patients [59], these values may not change in the same direction following treatment. For example, serum BDNF was increased after ECT sessions in TRD patients, while plasma BDNF remained unchanged [60], and the correlation between plasma and serum BDNF observed at baseline was lost after 6 weeks of treatment with antipsychotics [59]. 

In our study, plasma BDNF was increased in responders compared to nonresponders in TRD patients after BLT. Interestingly, in TRD patients following treatment with ketamine [61], ECT [52] or different atypical antipsychotic [62], plasma BDNF levels only increased in responders compared to nonresponders. Similarly, in TRD patients, plasma BDNF rose in responders and partial responders, but not in nonresponders to rTMS [63]. In another study in TRD patients, plasma BDNF even decreased, primarily in ECT nonresponders [64]. An early change in plasma BDNF after a single ketamine infusion was positively correlated with clinical improvement after 1–2 weeks of multiple ketamine infusions [65], while one study reported no correlation between plasma BDNF and the achievement of response or remission following ECT in TRD patients [66]. Since the aforementioned studies [61,62,66] did not include a control group, it is unknown if those patients had a lower plasma BDNF concentration at baseline. Therefore, the majority of trials [52,61,62,63,64], along with our results, suggest that plasma BDNF may be a marker of response to various interventions in TRD, and that changes in BDNF turnover may be a shared mechanism of many different TRD treatments, while our results further expand these findings to BLT. Similar outcomes were found in MDD patients [23], implicating a similar role of BDNF in response to antidepressants in both MDD episode and TRD.

In addition, in our study, plasma BDNF concentration was higher in patients who achieved remission, compared to nonremitters with TRD. Another line of evidence associated higher baseline BDNF with remission, showing that serum BDNF concentration at baseline was significantly increased in TRD remitted patients who received ECT compared to nonremitters [49,52,67], suggesting that increased baseline BDNF levels are required for patients to achieve remission. 

The increase in BDNF only in responders and remitters may be the consequence of symptom improvement, an increase in peripheral BDNF gene expression, and/or increase in brain BDNF levels. The beneficial effects of physical activity on BDNF are well known [68]. It might be that the BLT-induced improvement of symptoms, particularly the reduction in psychomotor retardation, the decrease in fatigue, and the improvement in motivation and increase in feeling of pleasure, resulted in increased patient activity. In addition, attending BLT treatment required frequent visits to hospital, which also required engagement in physical activity, and may have positively affected BDNF levels. However, the latter factor was less likely the explanation, given that all patients regularly received BLT, and the raise in BDNF was only detected in responders. While the effects of BLT on human BDNF gene expression is unknown, compelling evidence comes from preclinical studies. For example, rats subjected to bright light during 4 weeks experienced increased hippocampal BDNF expression [26,69], compared to the dim light [69] or control group [26], while, in turn, the exposure to dim light induced the decrease in hippocampal BDNF expression [69]. Similarly, bright light treatment in mice resulted in an increase in hippocampal mRNA BDNF expression and activated BDNF/TrkB signaling in comparison to dim light exposure [70]. A marginal correlation was found between the BDNF gene expression level in peripheral blood and the plasma level in the drug-naïve first-episode MDD group [42]. Despite such robust preclinical evidence on the bright light effects on hippocampal BDNF, it is still unclear how bright light affects plasma BDNF. There is some evidence on the correlation between BDNF plasma and brain tissue levels [71], and plasma BDNF levels were correlated with some hippocampal subfield volumes in first-episode MDD patients and healthy controls [44], and with cerebrospinal fluid BDNF [47]. These data might suggest that plasma BDNF levels indicate BDNF alterations in the brain [47,71]. However, other sources also contribute to plasma BDNF, such as muscles, vascular endothelium, platelets, and other blood cells [72].

### 4.3. Limitations and Strengths of the Study

Our study had no control group exposed to BLT, and the TRD group was not exposed to placebo; thus, the effects of BLT cannot be compared to other treatments or placebo, and therefore, placebo response cannot be ruled out. In fact, according to the recent meta-analysis, the magnitude of placebo response in TRD trials was large across different interventions [73], as was the placebo response in BLT trials in MDD. In the present study, our patients were recruited regardless on the season, but the improvement to BLT in nonseasonal depression was similar in autumn/winter and spring/summer periods, although the predictor of response to BLT in TRD patients was diurnal variations in mood, which was also not measured in our study [20]. However, seasonal variations were reported, at least in serum BDNF levels [20,29].

The present study also has strengths. It is the first study to measure plasma BDNF levels following BLT. It was sufficiently powered, and included a healthy control group, and homogenous comparison groups of MDD patients, separately analyzed as a first-episode and a recurrent-episode drug-free group. Of note, the minority of studies focusing on peripheral BDNF in TRD recruited the control group [30]. Only females were recruited, given previously reported sex differences in peripheral BDNF levels [74,75] and correlations between plasma BDNF and severity of symptoms evaluated with the HAMD-17 scores in female MDD patients [56]. Another reason is that daily variations in BDNF are not detected in female plasma [76]. In addition, all participants were recruited from the ethnically homogenous population, which may also be important due to previously observed differences in serum BDNF levels [77].

## 5. Conclusions

Our results revealed decreased plasma BDNF concentrations in patients with various forms of depression compared to healthy controls, and increased plasma BDNF levels associated with response and remission to BLT in TRD patients. Our findings also support the view that BLT may be considered a good clinical practice in patients with difficult-to-treat depression [78], but the more accurate role of BLT in TRD remains to be established in randomized trials. Given the association between the increase in plasma BDNF levels with the beneficial effects of BLT, which was previously reported across several other TRD treatments, BDNF-related parameters may be candidates for markers of treatment response in difficult-to-treat patients with depression.

## Figures and Tables

**Table 1:** Study procedures and groups.

Assessments	HC at Baseline	First Episode at Baseline	Recurrent Episode at Baseline	TRD at Baseline	TRD after 4 Weeks of BLT
Structured clinical interview				x	
HAMD-17, MADRS				x	x
Plasma BDNF concentration	x	x	x	x	x

HC = healthy control subjects; HAMD-17 = The Hamilton Depression Rating Scale-17; BLT = bright light therapy; MARDS = Montgomery–Åsberg Depression Rating Scale; TRD = treatment-resistant depression.

**Table 2:** Demographic and clinical data of subjects included in the study.

	HC (N = 71)	First Episode (N = 55)	Recurrent Episode (N = 25)	TRD (N = 54)	Statistics
Smokers (%)	33.3	29.1	28.0	38.9	χ^2^ = 13.18; *p* = 0.001
Positive family history (%)	NA	33.3	24.0	38.9	χ^2^ = 1.70; *p* = 0.427
The presence of life-time suicide attempts (%)	NA	3.6	8.0	9.3	χ^2^ = 1.46; *p* = 0.483
Age (years, median, min–max)	58 (37–68)	45 (19–67)	54 (19–78)	53 (21–69)	H = 21.61; *p* < 0.001
HAMD-17 scores (median, min–max)	NA	22 (17–37)	23 (17–32)	21 (16–27)	H = 2.80; *p* = 0.246
MADRS scores (median, min–max)	NA	25 (17–42)	27 (20–51)	27 (19–36)	H = 1.97; *p* = 0.373

HC = control subjects; TRD = treatment-resistant depression; HAMD-17 = Hamilton Rating Scale for Depression; MADRS = Montgomery–Åsberg Depression Rating Scale; NA = not available.

**Table 3:** The effect of BLT on MDD symptoms in patients diagnosed with TRD. Data are presented as median (min–max).

	TRD		Wilcoxon Test	
	Before Phototherapy	After 4 Weeks of Phototherapy	Z	*p*
HAMD-17 scores	21 (16–27)	10 (3–17)	6.41	<0.001
MADRS scores	27 (19–36)	13 (4–21)	6.40	<0.001

HAMD-17 = Hamilton Rating Scale for Depression; MADRS = Montgomery–Åsberg Depression Rating Scale; TRD = treatment-resistant depression.

## Data Availability

Data is unavailable due to privacy or ethical restrictions.
